# Spectral Identification of Disease in Weeds Using Multilayer Perceptron with Automatic Relevance Determination

**DOI:** 10.3390/s18092770

**Published:** 2018-08-23

**Authors:** Afroditi Alexandra Tamouridou, Xanthoula Eirini Pantazi, Thomas Alexandridis, Anastasia Lagopodi, Giorgos Kontouris, Dimitrios Moshou

**Affiliations:** 1 Agricultural Engineering Laboratory, Faculty of Agriculture, Aristotle University of Thessaloniki, 54124 Thessaloniki, Greece; tamouridoualex@gmail.com (A.A.T.); renepantazi@gmail.com (X.E.P.); 2 Laboratory of Remote Sensing and GIS, Faculty of Agriculture, Aristotle University of Thessaloniki, 54124 Thessaloniki, Greece; thalex@auth.gr (T.A.); giorgoskontouris@gmail.com (G.K.); 3 Plant Pathology Laboratory, Faculty of Agriculture, Aristotle University of Thessaloniki, 54124 Thessaloniki, Greece; lagopodi@agro.auth.gr

**Keywords:** plant pathology, MLP-ARD, disease detection, artificial intelligence, precision agriculture

## Abstract

*Microbotryum silybum*, a smut fungus, is studied as an agent for the biological control of *Silybum marianum* (milk thistle) weed. Confirmation of the systemic infection is essential in order to assess the effectiveness of the biological control application and assist decision-making. Nonetheless, in situ diagnosis is challenging. The presently demonstrated research illustrates the identification process of systemically infected *S. marianum* plants by means of field spectroscopy and the multilayer perceptron/automatic relevance determination (MLP-ARD) network. Leaf spectral signatures were obtained from both healthy and infected *S. marianum* plants using a portable visible and near-infrared spectrometer (310–1100 nm). The MLP-ARD algorithm was applied for the recognition of the infected *S. marianum* plants. Pre-processed spectral signatures served as input features. The spectra pre-processing consisted of normalization, and second derivative and principal component extraction. MLP-ARD reached a high overall accuracy (90.32%) in the identification process. The research results establish the capacity of MLP-ARD to precisely identify systemically infected *S. marianum* weeds during their vegetative growth stage.

## 1. Introduction

### 1.1. Weed Infestations

Weed infestations are considered a major impediment to global agricultural industry. Unfortunately, the current management policies rendered weed infestations increasingly problematic, particularly in arable crops [1]. Therefore, the application of chemicals as herbicidal agents proved to be inefficient. The main reason is the rising population of resistant weeds, mostly affecting arable crops.

Weed presence in cultivated areas can result in significant losses of crop quantity, percentages ranging depending on weed density [2]. When it comes to *Silybum marianum*, an efficient control approach requires a sufficient grasping of inter-specific relations of Silybum and the affected cultivated species. The recently developed bioeconomic weed management models do not contribute to an accurate estimation of yield loss. It was proposed that thistle presence leads to low nitrogen levels, resulting in low productivity in wheat crops by as much as 36% [3,4]. Thistle species, when predominantly infesting a cultivation, can severely impede crop production, resulting in a reduction of up to 47% in cases of spring wheat and grain sorghum [5,6].

### 1.2. Silybum marianum

*Silybum marianum* is a highly destructive thistle species, forming tall solid patches, preventing any other species developing by depriving them of necessary vital resources, including water and soil nutrients, especially pestering areas with fertile soil. Silybum propagates by forming flowerheads containing seeds, which carry pappi to facilitate their dispersion. Thus, thistle infestations persist through continuous cultivations.

Thistles grow in rosettes establishing competitiveness, resulting in low yield productivity. Silybum is becoming a serious and aggressive weed of wheat species (*Avena* sp., etc.) [7,8] and it also infests cattle grazing areas [9], resulting in toxicity incidents [10]. Thistles also degrade crops by attracting and hosting harmful pests [11].

### 1.3. Fungi as Bioherbicides

A bioherbicide is a targeted plant pathogen that can be utilized for weed management. It is based on discovering a host-specific pathogen from the targeted weed’s region of origin, ensuring the exclusivity of the pathogen’s infection. In various cases of weed biocontrol programs, smut fungi proved to be highly suitable [12]. Smut fungi are highly host-specific [13], to the point of exclusivity [14]. This is fundamental for using a bioherbicide in weed management [15].

**Figure 1:**
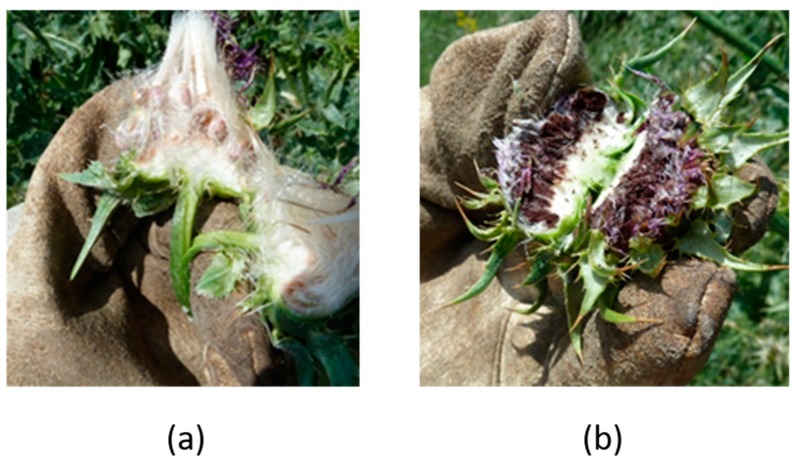
Effect of *Microbotryum silybum* infection. Section of a flowerhead produced by a healthy *Silybum marianum* plant (**a**) vs. a flowerhead produced by an infected *S. marianum* plant (**b**). The shape of the infected flowerhead is obviously stunted and the black color on the inside is caused by the teliospores of *M. silybum*.

Fungi of the Basidiomycetes class, order of Ustilaginales, are responsible for causing smut infections. Smut fungi cause intersystemic infections and their teliospores form in mass under the epidermis of the leaves or inside the flowerheads of their host, preventing the formation of the host’s own seeds in the second case. The fungus used in this research, *Microbotryum silybum*, belongs to smut fungi, and *S. marianum* is its natural host in Greece. The fungus is host-exclusive, being harmless to other plant species. It infects the host systemically, producing teliospore masses inside the flowerheads. The teliospores are in the form of fine dust, which are easily dispersed by wind. By forming teliospores inside the host’s flowerheads, the presence of the fungus immediately ensures its own dissemination and impedes the host’s propagation (Figure 1).

Due to the impediment of the infected weed’s propagation, the host specificity, and the intersystemic nature of the fungal infection, *M. silybum* is considered an excellent biocontrol agent for the case of *S. marianum*.

Diagnosis of the infection by external examination during the growth stages is impossible due to the absence of any external symptoms before the mature stage and flowerhead appearance and development. *Microbotryum silybum* is a key thistle biocontrol agent [16].

Disease recognition is considered crucial for applying weed biocontrol. Unfortunately, when it comes to intersystemic infections (more precisely, smut fungi), the lack of visible symptoms renders detection challenging. Previous research also focused on non-visible symptoms, also utilizing spectral analysis [17,18].

### 1.4. Neural Networks and Disease Recognition—MLP-ARD

Neural networks are universal approximators, and are able to describe any nonlinear function given enough hidden units and training data to train the neuron weights. Several classification algorithms are utilized in crop disease detection. In the multilayer perceptron (MLP) networks, the nodes process inputs from previous layers. Consequently, the information flow is in one direction, going to the output layer [19]. In a neural network model, the in-between neuronal units are related to the degrees of freedom the model possesses in terms of expressing the function embedded in data collections, and they define the representation ability of the model [20]. Adequate neuron units in processing are significant for recognizing imagery features.

The term artificial neural networks (ANN) refers to computational and machine learning techniques simulating the architecture of the functions of biological neural systems, providing solutions to complex nonlinear problems by classifying and labeling feature vectors [21]. The appropriate combination of abovementioned architectures and multi-sensors contributed to more accurate rates of assessing plant infestations. Extensive studies [22,23] proved optical data to be crucial for the infestation assessment and severity classification in plant leaves [24].

Numerous cucumber plant infections (e.g., downy mildew, anthracnose, and powdery mildew) were studied by Pixia and Xiangdong [25] by means of leaf imagery processing. Algorithms for data analysis, such as an ANN-based disease classifier and the Gabor filter were applied by Kulkarni and Patil [26], reaching a precision of 91%.

Pantazi et al. [27] applied machine learning techniques tackling the problematic discrimination between biotic and abiotic stress symptoms. Various self-organizing models achieved the assessment of canopy health status. More precisely, in the current study, hyperspectral imagery provided information concerning the discrimination between nitrogen-deprived and yellow rust-infested winter wheat plants.

A multilayer perceptron (MLP) comprises numerous layers. This type of architecture propagates feature data in a feed-forward way through the layers, and it can be calibrated in a way that minimizes the quadratic error [28]. In literature, there are many references of MLP applications for weed and crop management cases.

Tamouridou et al. [7] applied an MLP-ARD approach, mapping *S. marianum* patches by combing unmanned aerial vehicle (UAV)-based multispectral imagery with texture features achieving 99.54% correct recognition. Furthermore, Vianna et al. [29] experimented with 20 different MLP models regarding the utilization of imagery for late blight detection. The detection was accurate at a percentage of 97%.

### 1.5. Scope

The present study intends to prove accurate disease identification through examining plant parts that do not contain the fungus themselves. Furthermore, it is very important that a non-destructive method is applied allowing the in situ application of the suggested procedure to any kind of crop. The discrimination between healthy and infected plants was highly successful mainly due to variation of the spectra reflectance between the two categories that was caused by the influence of the fungus.

The precise identification of intersystemic infection by *M. silybum* is possible and is achieved via in situ spectroscopy. A previous study by Pantazi et al. (2017) [30] achieved similar classification by using hierarchical self-organizing maps (SOMs). In the current study, an MLP-ARD approach was employed for recognizing the presence of the *M. silybum* fungus in *S. marianum* plants. The algorithm was given the spectral reflectance curves taken from *S. marianum* leaves. The acquisition of spectral reflectance curves was completed on the study area with a portable spectrometer collecting spectral reflection values in the 400–1000 nm range of the spectrum. Source spectra underwent pre-processing for acquiring features necessary for the disease detection procedure. This identification of the *M. silybum* infection proves that it can be regarded as a practical and valuable biocontrol method, to be applied for *S. marianum* weed management, providing an alternative to yield loss or herbicide application dilemmas.

## 2. Materials and Methods

### 2.1. Study Area

**Figure 2:**
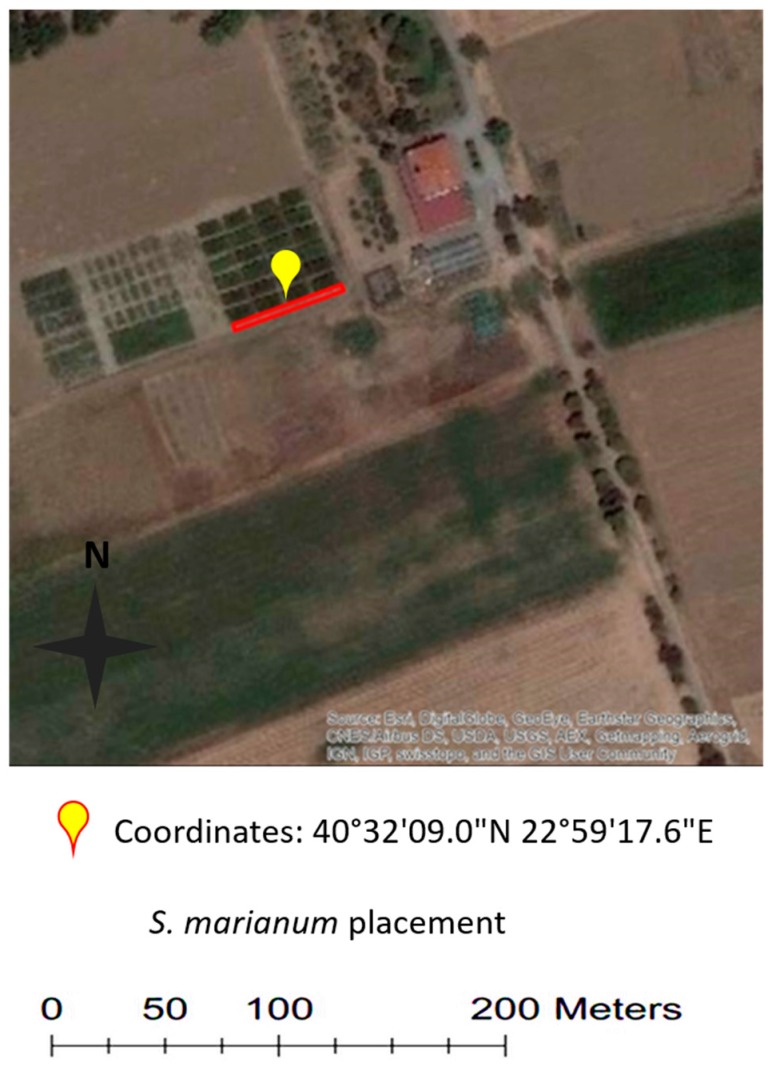
Study Area. The location of the *S. marianum* experimental set-up is depicted in the red rectangle.

The presented experiment took place in Thessaloniki, on grounds belonging to the Aristotle University of the Thessaloniki farm. The coordinates of the experimental location were 40°32′09.0′′ N, 22°59′17.6′′ E and the exact positioning of the plants is depicted in the red frame in Figure 2. The area’s ground topography is level, and the exact elevation is 3 m. The area’s climate is of the Mediterranean type, and the average annual temperature is 20 °C, with a temperature range of −5 to 43 °C depending on the season. There is an average annual precipitation of 450 mm, and the soil is organic and belongs to the silty clay type.

### 2.2. Plant Material Establishment

In the duration of the season preceding the presented study, *S. marianum* seeds were acquired from healthy plants and *M. silybum* teliospores were acquired from already diseased *S. marianum* heads, in order to be used in an artificial inoculation process.

Fourteen seeds were sowed during December in small containers, and kept indoors at 20 °C. After the germination of the seeds, the emerging rosettes were transplanted into larger containers, and were moved to a controlled environment enclosed section, with a temperature range of 5–25 °C. The pots remained in the greenhouse for 15 days. Successively, in the process of their acclimatization, the rosettes were placed in a non-heated greenhouse for four more days.

Subsequently, they were positioned outdoors at a location partially sheltered from the weather for three days. Finally, the young plants were planted in the aforementioned field location (Figure 2). The fourteen plants were randomly planted, 50 cm apart, in a linear-shaped area.

After the planting of the rosettes in the field, an artificial inoculation took place. A teliospore solution of approximately 5 × 106 teliospores/mL H_2_O was created and was applied via injection close to the apical meristem of seven rosettes. One milliliter of the solution was administered to each plant. To the seven rosettes of the control group, 1 mL of distilled water was applied via injection.

### 2.3. Data Acquisition

A spectrometer (described below) was utilized in order to conduct the measurements on two separate dates, 4.5 and 5.2 months (referring to the mathematical definition of the 30-day month) post seed germination. Both dates belonged to the same growth stage. The plants were fully developed, but not yet flowering. Measurements were conducted on three different leaves selected randomly. On each one of the leaves, six measurements were conducted on different locations on the leaf. The measurements were always taken from the upper side of the leaf. The measurement process was non-destructive. After carrying out the 18 total measurements for each *S. marianum* plant, a normalization procedure was performed with a white 99% spectralon.

The spectrometer utilized for the measurements was manufactured by PP Systems. It was a Unispec Single Channel (SC), with a visible and near-infrared (VNIR) sensor between 310–1100 nm, and a spectral sensitivity <10 nm, a diode array bin size of 3.3 nm, 0.1% repeatability, and a scan time which did not surpass 1 s (including integration time). Additionally, the absolute accuracy did not exceed 0.3 nm, and there was a 16-bit-type analog-to-digital (A/D) converter.

Furthermore, the integration time was 4–3200 ms, the light source consisted of an integral 7.0 W halogen bulb suitable for sampling under any light conditions. For the measurements, the leaf clip fore optic was utilized, taking point measurements. During the spectrometer calibration which took place before the beginning of the measurements, the integration time was determined and it remained consistent throughout the procedure.

### 2.4. Data Analysis

For the dataset construction, 104 signatures from infected *S. marianum* (half acquired 4.5 months post seed germination and the other half after 5.2 months) and 103 signatures from the control group (acquired 5.2 months post seed germination) were utilized. As a result, 207 spectra in total were retained for the calibration process of the prediction models.

From the aforementioned dataset, comprising 207 spectral signatures, 70% (145 signatures) were randomly selected for the network learning, while the other 30% (62 signatures) were employed for the validation process.

**Figure 3:**
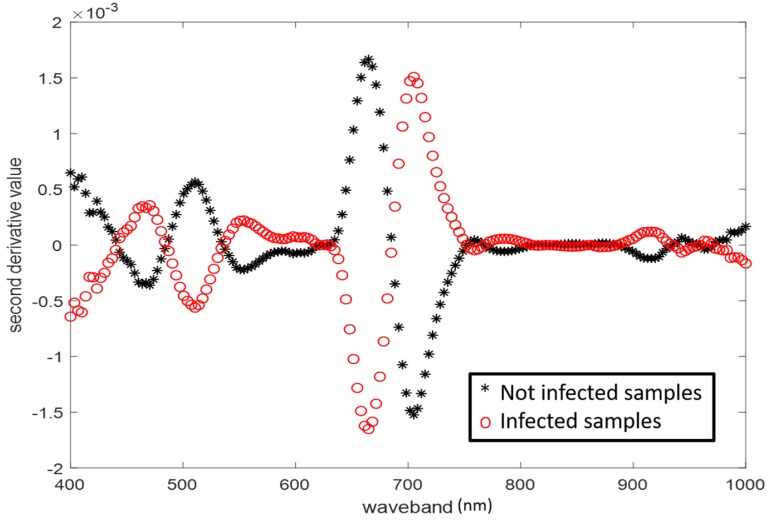
Mean second derivatives of spectra of diseased and healthy *S. marianum*.

In order to enhance spectral variances, the first and second derivatives were calculated from the spectral signatures. More precisely, second derivatives (Figure 3) were applied to simultaneously eliminate constant and linear background. Furthermore, the standard normal variate technique (SNV) was employed in order to achieve scatter removal [31]. SNV was also applied in order to center the spectral signatures on their mean values, and to eliminate the path-length variations by normalizing with the standard deviation.

Additionally, the transformed spectra were subjected to principal component analysis (PCA). PCA is a statistical method, widely employed for the processing of large multidimensional groups of data. The main axes of the data deviation are located via the principal components, and the data are placed in the coordinate system determined by two or more PCs [32]. The number of coherent variations in datasets is fairly lower than the amount of original variables. Therefore, PCA achieves a projection of the dataset elements in a space with fewer dimensions [33]. The number of principal components was determined by selecting the number of components that performed best in the classification by iterating from 1 to 15. In the direction of building a robust prediction algorithm for disease identification, the first 10 principal components’ scores were reserved to keep the maximum amount of information.

### 2.5. MLP-ARD Classifier

Bishop [34] developed the MLP-ARD architecture which adds regularization to the MLP model. The MLP-ARD model achieves weight vector normalization by taking into account weight classes. For every weight, there is a weight decay hyperparameter, corresponding to predictive variables and to hidden neuron units.

The MLP-ARD architecture that was used consisted of 10 input neurons corresponding to the 10 principal components, 15 hidden neurons, and two output neurons corresponding to two classes, healthy and infected. The model was trained for 1000 iterations. The described model was used to classify the 145 *S. marianum* spectral signatures. Accuracy assessment was carried out through consideration of the confusion matrices on the validation dataset of 62 spectral signatures. 

## 3. Results

The classification results are presented in Table 1.

**Figure 4:**
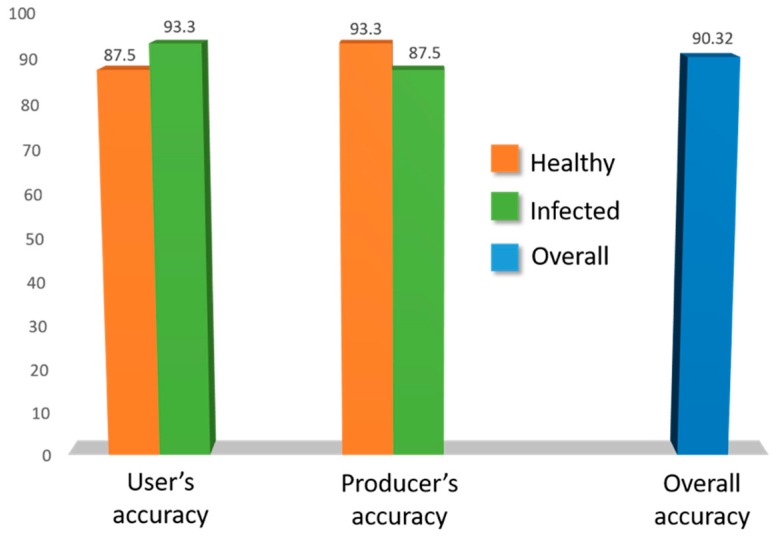
Accuracy of multilayer perceptron/automatic relevance determination (MLP-ARD) classification. In this figure, both user’s and producer’s accuracy percentages are depicted, with the orange color for healthy and green for infected *S. marianum* plants. The overall accuracy percentage is depicted in blue.

Performance evaluation was carried out through the contingency matrices from validation data, which consisted of 62 signatures (30 from infected and 32 from control plants). The results of the assessment and percentages of user’s, producer’s, and overall accuracies are illustrated in Figure 4.

**Figure 5:**
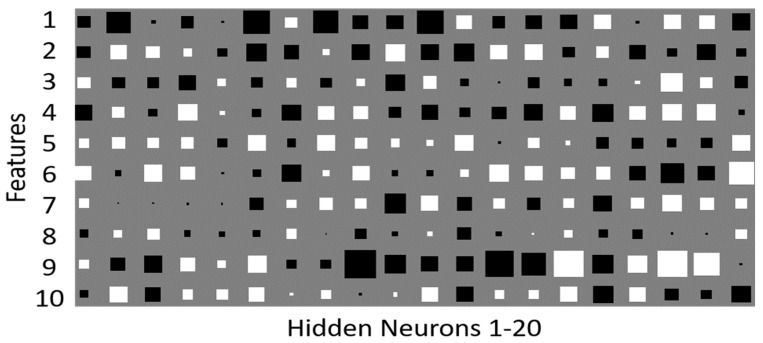
Hinton diagram of the trained MLP-ARD algorithm. The X-axis represents the hidden neurons and the Y-axis represents the input features.

Regarding the performance of MLP-ARD classifiers, in order to visualize its function, the below Hinton diagram was created depicting the component weights of the hidden neurons (Figure 5).

The Hinton diagram illustrates the weight values connecting the 10 spectral components and 20 hidden neurons. The white squares represent the positive weight values, while the black squares represent the negative ones. The algorithm suppressed the weights of the less important characteristics while enforcing the weights of the more active ones, considering the size of each square which represented the relative size of the weight. The state of the individual features can be derived by the Hinton diagrams. 

Figure 5 illustrates the way that the MLP algorithm influenced the weight distribution. It appears that the larger weights corresponded to the first and ninth input feature, while the smaller ones corresponded to the eighth inversely with the hyperparameter values. The ARD procedure regularized the weight groups corresponding to each feature in such a way that the most active weight groups corresponding to the most relevant features received a greater update, resulting in higher values for these weights at the end of training procedure. To the contrary, the less important weights associated with less relevant features received less updates, so they had lower values. This means that they did not affect the classification result in any significant manner.

A quantification of the influence of the individual features could be derived by observing the L2 norms of the weights and the relevant values of the hyperparameters (α) that could be calculated using Equation (1).

(1) $$F(\omega )=G+\sum_{k}a_{k}Ew_{\left(k\right)},$$

where $Ew_{\left(k\right)}=\frac{1}{2}\sum {\omega^{2}}_{j}$, ${\omega \;}_{j}\in R$ represents the weights and j weight indicator of the class *W_(k)_*.

**Figure 6:**
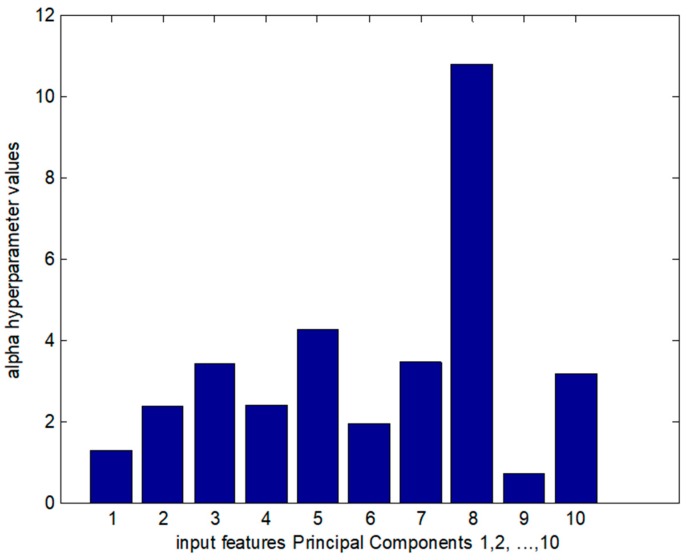
The 1–10 alpha hyperparameters are illustrated.

At the later stage of the training procedure, higher norms are connected to lower hyperparameter values that, in turn, are connected to higher weight variance. In Figure 6, the hyperparameter values for each weight group could be calculated for connected individual input features with hidden layer units. The alpha hyperparameters behaved in exactly the opposite way to the weight groups of the Hinton diagram. This means that the larger the weight that connected the feature, the smaller the associated hyperparameter was. This led to the process of soft selection of the more active weight groups based on the magnitude of the hyperparameter, leading to small values that converted to zero for the hyperparameter connected to the most important input feature. This was observed in the case of very low values of hyperparameters associated with the input features of the first and ninth principal components.

**Figure 7:**
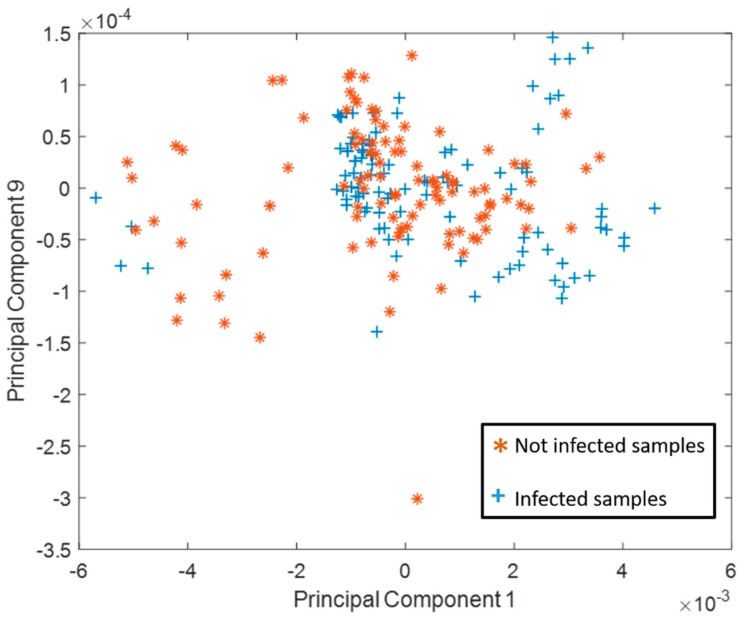
Principal components 1–9. The values of the two principal components obtained for the 207 spectral signatures.

An analysis of the first and ninth principal component displayed the most active results during training for both healthy and diseases spectral signatures. The size of the two principal components for each of the signatures could be derived from Figure 7. The two principal components covered almost 76% of the overall spectral variance. More precisely, the first principal component demonstrated a variance of 75.9%, and the ninth demonstrated a variance of 0.1%. The infected samples in the principal component space illustrated in Figure 7 showed a wider distribution than the healthy ones.

## 4. Discussion

MLP-ARD reached high accuracy (90.32%) and its reliability was confirmed by the high percentage and the K hat statistic (0.807). The reflectance curve similarity between infected and healthy *S. marianum* plants was responsible for the increased user’s accuracy performance. User’s accuracy for the infected plants was lower than the producer’s, since the dispersed nature of symptoms (which the calibration dataset could not capture) led to the variability of test spectra.

**Figure 8:**
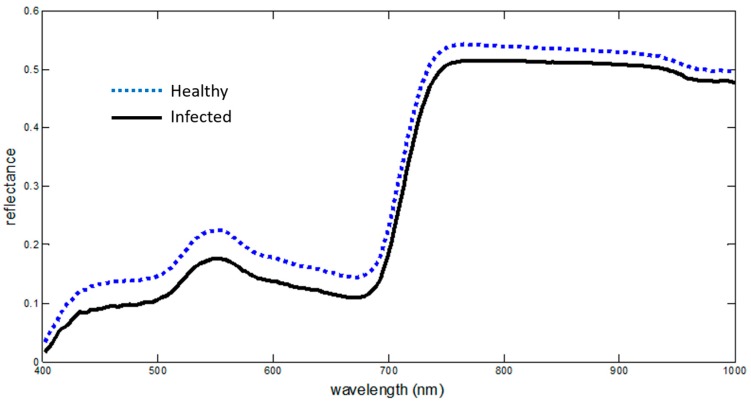
Reflectance curves. The average of the reflectance values acquired from the infected *S. marianum* plants is depicted with a solid black line, and the average of the reflectance values acquired from the healthy *S. marianum* plants is depicted with a dotted blue line. The horizontal axis represents the wavelength regions, and the vertical axis represents the reflectance values.

Despite the absence of any obviously discernible symptoms on the infected *S. marianum* leaves, the spectral behavior of the reflectance curve in the Visual and Near InfraRed (VNIR) range decisively rendered the infected plants detectable. Previous studies employing a hierarchical Self-Organising Map (SOM) classifier, called XYF on the same dataset, achieved a higher classification rate of 95.16% (Pantazi et al. 2017) [30]. Although visible symptoms are not present throughout the progress of systemic infections, several studies showed that the presence, development, and metabolic procedures of the pathogen affected the optical properties of the plant organs, thus resulting in a modified spectral signature. The changes may vary in scale and in characteristics; however, it is not these features, but rather the change itself that signifies the differentiation between the two types of spectral signatures in this case. Lorenzen and Jensen [35] studied the reflectance changes caused by fungal infection on cereal leaves, and found it increased in the visible region. On the other hand, a similar study by Ausmus and Hilty [36] showed considerably lower NIR reflection on infected plants. The present research detected that an analogous reduction in the reflectance in the VNIR spectrum was in the spectra of the diseased thistles (Figure 8).

Malthus and Madeira [37] employed high resolution spectroradiometry in order to examine the reflectance changes caused on *Vicia faba* fungus-infected leaves. Their observations concluded that the leaves’ spectral reflectance had constantly similar values in the visible area of the spectrum, whereas their near-infrared shoulder reflectance (800 nm) declined. The findings can be attributed to the leaf cell structure collapse caused by the fungal activity. Additionally, there was a close association between the first-derivative reflectance spectra, those of the visible spectrum, and the infection severity.

The significance of smut fungi is that, during the stage of vegetative growth, the infection is only present in the plant’s apical meristem. Hence, neither alteration of the pigment concentration, nor leaf cell structure collapse takes place, a situation that hinders the identification of the diseased plants.

In the case of *Silene dioica*, the fungus, *Microbotrium violaceum*, does exist within the specific plant tissues that were examined with the employment of NIR spectroscopy; thus, the detected spectral alteration was explained by chemical alteration occurring because of the chemical structure of the fungus or the host plant’s reaction to it [38]. In addition, in that research, the spectral signatures were acquired in laboratory conditions employing a destructive method (using dried and milled leaves).

Due to major concerns raised on the issue of food safety and environmental hazards, there is a shift toward minimizing the application of chemicals in agriculture, and to employing alternative methods of weed control. In regards to alternative weed management methods, biological weed control is environmentally responsible and suitable to be applied in a variety of circumstances [39].

*M. silybum* is a smut fungus that can serve as a key bioherbicide for *S. marianum* weeds [16]. Its determined efficiency against *S. marianum* can establish its use as an agent in biological control applications.

The confirmation of the weeds’ infection through field testing during the growing season will determine whether the biological control application is sufficient, or whether further action, and to what extent, needs to be taken for weed control. The abovementioned knowledge could be integrated in a decision support system (DSS), leading to the introduction of biological weed control in an overall environmentally friendly cultivation system.

The precise identification of successfully infected *S. marianum* allows for accurate mapping of non-infected spots, enabling the assessment of the effectiveness of biological control. In the opposite scenario of plant pathology, the generalization potential of the developed classification model enables the precise identification of systemically infected plants to allow the accurate mapping of diseased patches in a crop field. The overall accuracy of the infection’s identification (90.32%) can be considered very satisfactory given the variability of the spectra of especially the healthy, but also the infested *S. marianum* plants. This identification of fungal presence enables the targeted and site-specific application of fungicides. All things considered, the precise application of herbicides, fungicides, or pesticides in general, can support the orthological and targeted chemical application and sustainable crop management.

## 5. Conclusions

The current study established that it is feasible to classify systemically infected and non-infected *S. marianum* plants by the smut fungus *M. silybum*. The discrimination was accomplished during the stage of vegetative growth through the utilization of the first 10 principal components derived from spectral signatures. The spectral signature acquisition was carried out in situ with a VNIR portable spectrometer. 

The classification results of the MLP-ARD algorithm were found to be highly accurate (overall accuracy of 90.32%) and reliable (K hat 0.807) through evaluation done using an independent validation dataset. Detection of *M. silybum* infection on *S. marianum* plants was proven to be feasible with the use of MLP-ARD. The presented research could set the grounds for various applications of precision agriculture, for example, the quick and convenient evaluation of biocontrol endeavors and assessment of innovative automatic-decision-based practices directed at pest eradication.

## Figures and Tables

**Table 1:** Contingency table of the multilayer perceptron/automatic relevance determination (MLP-ARD) network results prediction (percentage of correct classifications).

	Network Prediction (Estimated Class)	
Actual Observations (Ground Truth)	Samples from Diseased Plants	Samples from Healthy Plants
Infected	28	2
Healthy	4	28
